# Effect of intravenous dexmedetomidine on sensory block duration in spinal anesthesia for lower limb surgery: a randomized controlled trial

**DOI:** 10.1016/j.bjane.2025.844672

**Published:** 2025-08-27

**Authors:** Simran Chahal, Anju R. Bhalotra, Rahil Singh, Shweta Dhiman, Snigdha Singh

**Affiliations:** Maulana Azad Medical College and associated Lok Nayak Hospital, Department of Anesthesiology, Bahadur Shah Zafar Marg, India

**Keywords:** Dexmedetomidine, Postoperative analgesia, Sensory block, Spinal anaesthesia

## Abstract

**Study objective:**

To study the effect of Intravenous (IV) dexmedetomidine during spinal anesthesia on duration of sensory block and postoperative analgesia in patients undergoing lower limb orthopedic surgery.

**Design:**

Prospective randomized double blind controlled trial.

**Intervention:**

Patients in intervention (DX) group received 0.5 mcg.kg^-1^ IV dexmedetomidine over 10 min. Spinal anesthesia was administered and an infusion of dexmedetomidine 0.5 mcg.kg^-1^.h^-1^ was given throughout surgery.

**Measurements:**

Onset time of sensory and motor block, maximum height of sensory block and duration of sensory and motor block were assessed. Intraoperative Heart Rate (HR), Blood Pressure (BP), Peripheral Oxygen Saturation (SpO_2_), sedation scores, postoperative pain scores, time to requirement of first analgesic and analgesic consumption over first 24h were noted.

**Results:**

Data of 58 ASA I/II adults was analyzed. Duration of sensory block, defined as time to two-dermatome regression, was 137.03 ± 25.02 min in DX group and 79.45 ± 11.27 min in the NS group (p = 0.000). Onset of sensory and motor block and maximum height of sensory block were similar. Postoperative VAS scores were lower in the DX group at 4h and 24h (p = 0.001, p 0.0001) and comparable at 0h, 8h and 12h. Time to requirement of postoperative analgesia was longer in the DX group (p < 0.001) and requirement of postoperative analgesics was higher in the NS group. Sedation scores and incidence of bradycardia were higher in the DX group, but hypotension was similar.

**Conclusion:**

IV dexmedetomidine (0.5 mcg.kg^-1^ followed by 0.5 mcg.kg^-1^.h^-1^) resulted in extended sensory and motor block, prolonged postoperative analgesia and reduced postoperative analgesic consumption with minimal side effects.

## Introduction

Lower limb orthopedic surgeries encompass a wide range of procedures which can be performed under neuraxial or regional anesthesia. Various modalities have been used to manage postoperative pain after lower limb orthopedic surgery. Opioids like fentanyl, morphine and tramadol have great analgesic potential and are commonly used for intraoperative and postoperative analgesia. IV administration of non-steroidal anti-inflammatory drugs is also utilized for the same purpose. Epidural analgesia provides effective pain relief and the option for continuous postoperative analgesia. Lower limb nerve blocks enhance pain management, facilitate early mobilization and improve patient satisfaction during recovery. However, all these modalities are associated with their own specific set of advantages and disadvantages.

Spinal anesthesia is commonly used for lower limb surgeries as it is easy to administer, has a rapid onset of action, and avoids the difficulties associated with airway management. When local anesthetic agents are used alone for spinal anesthesia, they provide a finite duration of analgesia. The duration of spinal analgesia and anesthesia can be prolonged by the addition of adjuvant drugs such as opioids, alpha-2 agonists, adrenaline, etc. to the local anesthetic or by placing a catheter.^1^^,^^2^

The synergistic effect of local anesthetics and alpha-2 agonists may be due to their differing sites of action. While local anesthetics block voltage-gated sodium channels which are necessary for nerve impulse transmission, alpha-2 agonists provide analgesia by several mechanisms at spinal and supraspinal sites. By binding to alpha-2 receptors in the spinal cord and brainstem, they inhibit transmission of nociceptive impulses through the posterior horn of the spinal cord, inhibit the release of norepinephrine, which is a neurotransmitter involved in transmitting pain signals, activate descending pain control pathways and promote the release of acetylcholine from spinal interneurons, resulting in increased synthesis and release of nitric oxide which may be involved in regulation of analgesia. They also modulate pain processing in the brain, making pain appear less intense and unpleasant, and the sedation and anxiety provided further help reduce the perception of pain.^3^^,^^4^ All these effects may contribute to prolonging the sensory block of spinal anesthesia. The prolongation of motor block of spinal anesthesia may be due to binding of these drugs to motor neurons in the dorsal horn.^5^ However, potential adverse effects like hypotension and bradycardia must be taken into consideration when these drugs are administered.

When administered IV, dexmedetomidine provides excellent analgesia without respiratory depression and has been safely utilized as a preoperative sedative or medication for patients undergoing surgery under regional anesthesia.^6^ A few earlier studies have reported a prolongation of the sensory block of spinal anesthesia by pre-administration of IV dexmedetomidine. The target site of IV administered alpha-2 agonists is also the alpha-2 receptors and a similar potentiation of spinal anesthesia may be achieved by administering the drug by the IV route. As most spinal additives have not obtained approval for intrathecal administration from regulatory bodies, it seems safer to opt for IV administration if a similar benefit can be obtained.

Kaya et al.^6^ found a significant increase in the duration of the sensory blockade of spinal anesthesia when patients received 0.5 mcg.kg^-1^ IV dexmedetomidine before spinal anesthesia with 0.5% heavy bupivacaine. Kavya et al.^7^ administered 0.5 mcg.kg^-1^ IV dexmedetomidine as a pre-spinal bolus, followed by a continuous infusion of 0.5 mcg.kg^-1^ for 1h and found a prolongation of both the sensory and motor blockade of spinal anesthesia.

While Harsoor S et al.^8^ and Bhirud PH et al.^9^ studied the characteristics of spinal anesthesia after the administration of 0.5 mcg.kg^-1^ IV dexmedetomidine before spinal anesthesia followed by an IV infusion of 0.5 mcg.kg^-1^.h^-1^ through the entire duration of surgery, they did not study the postoperative pain scores and analgesic requirements. It has been suggested that the use of IV dexmedetomidine intraoperatively may result in lower postoperative pain and reduced opioid consumption,^10^ and improvement in the quality of recovery and chronic pain after surgery.^11^

We hypothesized that the administration of 0.5 mcg.kg^-1^ IV dexmedetomidine before spinal anesthesia followed by an IV infusion of 0.5 mcg.kg^-1^.h^-1^ throughout surgery should result in prolongation of the sensory block of spinal anesthesia, prolonged postoperative analgesia and reduced postoperative analgesic consumption with minimal side effects. Our primary objective was to compare the duration of sensory blockade of spinal anesthesia with and without IV dexmedetomidine bolus and infusion in patients undergoing lower limb orthopedic surgery. Secondary objectives were to compare the other characteristics of spinal anesthesia and postoperative pain and analgesic requirements.

## Methods

### Ethics approval

This prospective, randomized, double-blind controlled trial was conducted between July 2023 and August 2024. The study protocol was approved by the Institutional Ethics Committee on 19/04/2023 and the trial was prospectively registered under the Clinical Trials Registry of India (CTRI/2023/06/054571) on 30/06/2023. The trial adheres to the principles of the Declaration of Helsinki. Written and informed consent was obtained from all participating patients. This manuscript adheres to the Consolidated Standards of Reporting Trials (CONSORT) guidelines for randomized controlled trials. The full trial protocol and statistical analysis plan are available on request.

### Inclusion and exclusion criteria

We studied adults of American Society of Anesthesiologists (ASA) physical status I/II of ages 18‒65 years of either sex scheduled to undergo elective lower limb surgery of an anticipated duration of 1‒2h under spinal anesthesia. Patients with contraindications to spinal anesthesia or previous failed spinal anesthesia, long-standing diabetes mellitus, cardiac or neurological disease, neuropsychiatric disorders, hypersensitivity to study drug, chronic treatment with opioids/sedatives and pregnant women were excluded from the study. Obese patients (BMI > 30 kg.m^-2^) were also excluded due to their higher risks of underlying cardiovascular comorbidities and consequent hemodynamic instability and altered drug pharmacokinetics.

### Randomization and Group allocation

Prospective patients were screened for eligibility and included in the study on the morning of surgery. Randomization was done by computer-generated numbers and allocation into groups by opening a sealed opaque envelope before surgery by an anesthesiologist not involved in the study protocol. Patients were randomly allocated in 1:1 to either Group DX (Dexmedetomidine) to receive IV dexmedetomidine bolus followed by dexmedetomidine infusion or Group NS (Normal Saline) to receive IV normal saline bolus followed by NS infusion through the duration of surgery.

### Blinding

Patients were blinded to their group allocation. All study drugs were prepared by an independent anesthesiologist who was not involved in the subsequent conduct of the study. Intraoperative and postoperative assessments, including pain scoring, were performed by a separate anesthesiologist who remained blinded to group allocation throughout the study.

### Anesthesia technique

A detailed pre-anesthetic check-up including history, physical examination, and investigations as indicated was carried out in all patients and the anesthetic procedure was explained. During the preoperative visit, patients were taught how to grade their pain using the Visual Analogue Scale (VAS).^12^ All patients were fasted as per ASA guidelines. Premedication in the form of tablet alprazolam 0.25 mg was given at night and at 6 am on the day of surgery.

In the operating room, the patients were placed in the supine position, and standard ASA monitoring consisting of ECG, non-invasive Blood Pressure (BP), pulse oximetry and temperature monitoring was instituted. Baseline Systolic Blood Pressure (SBP), Diastolic Blood Pressure (DBP), Mean Arterial Pressure (MAP), Heart Rate (HR), and Peripheral Oxygen Saturation (SpO_2_) were recorded. IV access was secured with a 16 G/18 G IV cannula and ringer lactate infusion was commenced at 20 mL.kg^-1^.h^-1^.

Patients in Group DX received 0.5 mcg.kg^-1^ IV dexmedetomidine in 20 mL of NS over 10 min (bolus dose). Spinal anesthesia was then administered and an IV infusion of dexmedetomidine 0.5 mcg.kg^-1^ in 20 mL of NS was given at 20 mL.h^-1^ through the duration of surgery. Patients in Group NS received 20 mL of NS IV over 10 min (bolus dose). Spinal anesthesia was then administered and an IV infusion of NS at 20 mL.h^-1^ was continued through the duration of surgery.

After receiving the bolus dose of the study drug in a volume of 20 mL over 10 min, the patient was assisted into the sitting or lateral position and using full asepsis, subcutaneous infiltration with 1 mL of 2% xylocaine was done at the L3‒L4 space. A subarachnoid block was performed using a 25-gauge spinal needle through the midline approach. After attaining free flow of CSF, 2.5 mL of 0.5% hyperbaric bupivacaine was administered intrathecally and following this, the patient was returned to the supine position immediately with the table maintained horizontally. Infusion of the study drug was then commenced in all patients as per group allocation at the rate of 20 mL.h^-1^ and was continued up to the last skin suture. All patients were given oxygen at the rate of 5 lpm via a face mask throughout the procedure.

At the end of the surgery, the patients were shifted to the Post Anesthesia Care Unit (PACU). On arrival in PACU, they received injection Paracetamol 1g IV and then henceforth every 8 hours. Pain scores using VAS were assessed at rest at 0h, 4h, 8h, 12h, and 24h. Patients with a VAS score > 3 received injection diclofenac 75 mg IV and if the patients were not relieved of pain within 30 min, they were given injection tramadol 1‒2 mg.kg^-1^ IV along with injection ondansetron 4 mg. If the patient was sleeping comfortably, the VAS score was considered to be zero. The duration of analgesia was taken to be the time from administration of subarachnoid block to the request of first additional post-operative analgesia or VAS > 3, whichever was earlier. The duration of analgesia and total additional analgesic consumption in 24h postoperatively was noted. Both the patient and the anesthesiologist following up the patient were blinded to the patient’s group allocation.

During the study period, hypotension, defined as a decrease in MAP below 20% of baseline or systolic pressure < 90 mmHg was treated with lactated ringer’s solution 200 mL over a 5 min period and then intravenous ephedrine 5 mg if the hypotension persisted. Bradycardia (HR < 50 bpm) was treated with intravenous atropine 0.5 mg. Only if the bradycardia and hypotension persisted or worsened, the study drug infusion would be interrupted until the vital signs stabilized.

### Measurements and data handling

The time of intrathecal injection was recorded. Sensory blockade was assessed using pinprick in the mid axillary line on both sides. The motor block was assessed immediately after sensory block assessment using a Modified Bromage Scale. Sensory and motor block were assessed every 2 min till the maximum height of the sensory block was attained and a Modified Bromage scale score of 3 was attained and thereafter every 10 min.

The onset time of sensory blockade was defined as the interval between intrathecal administration and the attainment of T10 sensory dermatome blockade. The maximum height of the sensory block achieved was noted. The duration of the sensory blockade was defined as the interval from intrathecal administration to the point of a two-dermatome regression of sensory block from the maximum level. The onset time of motor blockade was defined as the time from intrathecal injection to Modified Bromage score of 3. Motor block duration was defined as the time from intrathecal administration to the point at which the Modified Bromage score was back to zero. After administration of the subarachnoid block, HR, SBP, DBP, MAP and SpO_2_ were recorded every 5 min for the initial 30 min, and then every 10 min for the duration of surgery. The Ramsay Sedation Score^13^ was used to score sedation every 10 min intra-operatively and upon arrival in the PACU. Excessive sedation was defined as a score greater than 4/6. The presence of any other complications in the perioperative period like nausea or vomiting and headache were also noted. At the end of the study, participants were asked to guess their group allocation (intervention, control, or unsure) as part of the blinding assessment.

### Primary and secondary outcomes

The primary outcome measure was the time for two-segment regression of sensory blockade of spinal anesthesia. Secondary outcomes were onset time of sensory block, onset time and duration of motor block, maximum level of sensory block achieved, postoperative pain scores, time to requirement of first additional postoperative analgesic and additional analgesic consumption over the first 24h.

### Sample size calculation

The primary outcome variable was the duration of the sensory block, defined as the time (in mins) required for two-segment regression following spinal anesthesia. Based on a previous study,^7^ the mean (± SD) regression time was 166.2 ± 26.7 min in the IV dexmedetomidine (bolus plus infusion) group and 133.2 ± 28.2 min in the normal saline group. Accordingly, an expected mean difference of 33 min and a pooled standard deviation of 28 min were used for sample size estimation. The sample size was calculated using the formula for comparing two independent means, assuming a Type I error (α) of 0.05 and a power (1 – β) of 80%, which yielded a requirement of approximately 22 participants per group. To account for a potential 30% dropout rate, the total sample size was increased, and 58 patients were enrolled. This calculation assumed a continuous, normally distributed outcome variable and was based on the referenced study.^7^

### Statistical analysis

Data was collected and analyzed using Statistical Package for Social Sciences (SPSS) version 24.0 (IBM Corp., Armonk, NY, USA). A total of 58 patients were included in the final analysis. Continuous variables, including duration of sensory block and time to regression, were summarized as mean ± standard deviation. The Kolmogorov-Smirnov test was used to assess the normality of data distribution, and the data were found to be normally distributed. Group comparisons for continuous variables were performed using the independent samples *t*-test. Categorical variables were expressed as frequencies and percentages, and comparisons between groups were made using the Pearson Chi-Square test. Pain and sedation scores, expressed as median [Interquartile Range, IQR], were compared between groups using the Mann-Whitney *U* test. For secondary endpoints, adjustment for multiple comparisons was applied to control the family-wise error rate, using the Bonferroni correction. A p-value < 0.05 was considered statistically significant.

## Results

**Figure 1:**
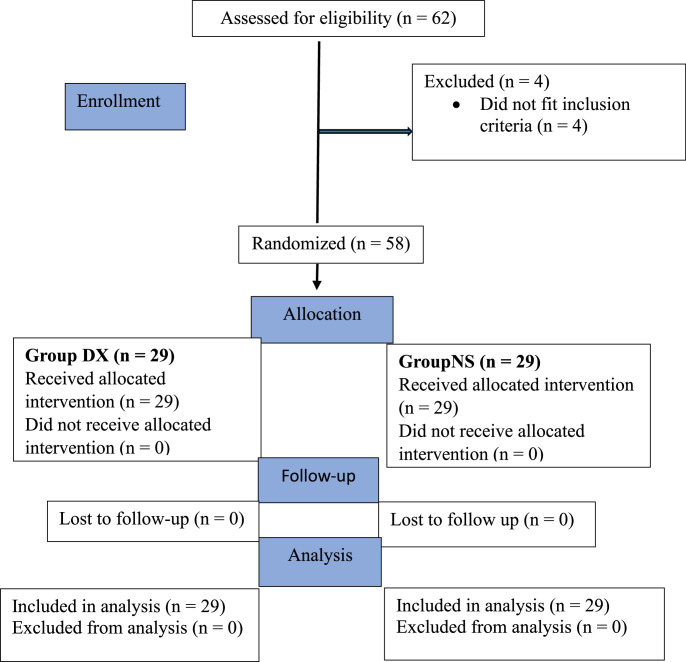
CONSORT flowchart of subject enrollment. CONSORT, Consolidated Standards of Reporting Trials.

Sixty-two patients were assessed for eligibility. Four patients did not meet the inclusion criteria. Fifty-eight patients were included in the study and their data analyzed (Fig. 1).

No differences in baseline patient characteristics were observed between the study groups (Table 1).

**Table 1:** Demographic data.

Parameter	Group DX	Group NS	p-value
Age (yrs)	35.00 ± 10.76	36.24 ± 13.18	0.696^b^
Sex ‒ Male/Female	22/7	24/5	0.517^a^
Height (cm)	162.72 ± 7.61	164.90 ± 7.39	0.275^b^
Weight (kg)	64.69 ± 10.20	69.00 ± 10.23	0.114^b^
BMI (kg.m^-2^)	24.30 ± 2.60	25.37 ± 2.85	0.140^b^
ASA grade I/II	21/8	16/13	0.172^a^
Duration of surgery (min)	80.34 ± 23.37	73.10 ± 20.76	0.217^b^
Duration of anesthesia (min)	117.93 ± 24.11	112.07 ± 21.77	0.335^b^

Data is expressed as Mean ± SD or as number of patients.

SD, Standard Deviation; BMI, Body Mass Index; ASA, American Society of Anesthesiologists.

a Using χ^2^ test.

b Using *t*-test.

**Figure 2:**
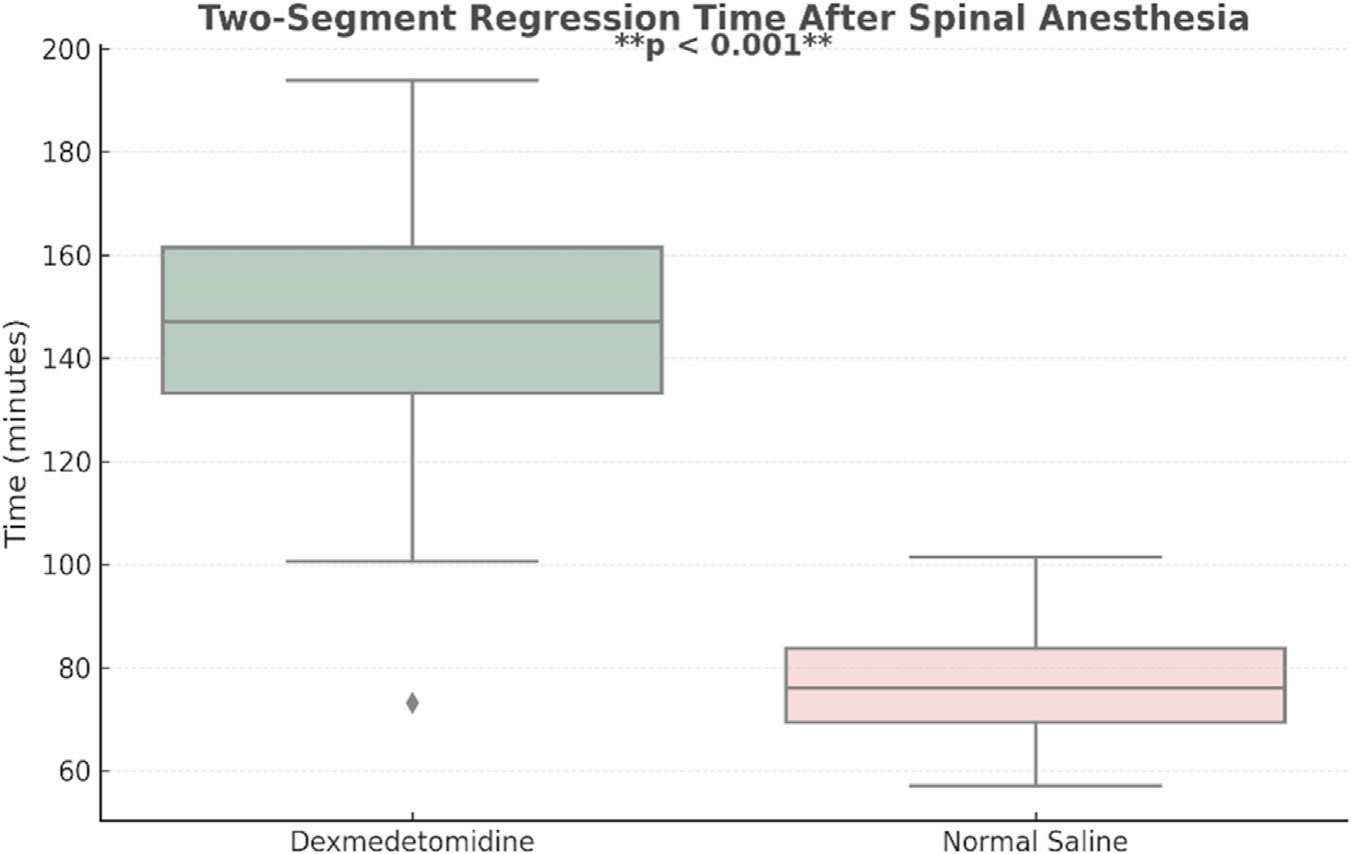
Boxplot showing two-segment regression time in the DX and NS groups.

The time for two-segment regression of sensory blockade was significantly prolonged in the DX group (137.03 ± 25.02 min) compared to the NS group (79.45 ± 11.27 min). An independent sample Student's *t*-test showed that this difference was statistically significant (p < 0.001). The calculated effect size (Cohen’s d ≈ 2.97) indicated a very large treatment effect, suggesting not only statistical but also strong clinical relevance (Fig. 2, Table 2).

**Table 2:** Primary and secondary outcomes.

Parameter	Group DX (n = 29)	Group NS (n = 29)	p-value
Onset time of sensory block (min)	3.24 ± 1.12	3.59 ± 1.12	0.246^a^
Onset time of motor block (min)	4.28 ± 0.88	4.55 ± 1.06	0.285^a^
Duration of sensory blockade (min)	137.03 ± 25.02	79.45 ± 11.27	< 0.001^a^
Duration of motor blockade (min)	236.34 ± 37.16	158.69 ± 23.64	< 0.001^a^
Maximum height of block achieved			
T6 dermatome	20	14	
T8 dermatome	8	12	0.239^b^
T10 dermatome	1	3	
VAS Scores			
VAS (0h/arrival in PACU)	0 (0‒0)	0 (1‒0)	0.102^c^
VAS (4h)	1 (2‒0)	2 (2‒1)	0.001^c^
VAS (8h)	1 (2‒1)	2 (2‒1)	0.051^c^
VAS (12h)	2 (2‒1)	2 (2‒1)	0.333^c^
VAS (24h)	0 (1‒0)	1 (2‒1)	0.0001^c^
Time to requirement of first postoperative analgesic			
≤ 4h	1	6	
> 4h to ≤ 8h	2	18	< 0.001^b^
> 8h to ≤ 12h	8	3	
> 12h to ≤ 24h	8	0	
> 24h	10	2	
Postoperative Diclofenac requirement			
0 dose	10	2	
1 dose	15	4	< 0.001^b^
2 doses	4	15	
3 doses	0	8	
Postoperative Tramadol requirement			
0 dose	26	19	0.027^b^
1 dose	3	10	

Data is expressed as Mean ± SD, Median (IQR) or numbers.

VAS, Visual Analogue Scale, T6, 6^th^ thoracic sensory dermatome, T8, 8^th^ thoracic sensory dermatome, T10, 10^th^ thoracic sensory dermatome.

a Using *t*-test.

b Using χ^2^ test.

c Using Mann-Whitney nonparametric test.

The onset of sensory blockade was 3.24 ± 1.12 min in the DX group and 3.59 ± 1.12 min in NS group (p = 0.246). The onset of motor blockade was 4.28 ± 0.88 min in the DX group and 4.55 ± 1.06 min in the NS group (p = 0.285). The duration of motor blockade was longer in the DX group (236.34 ± 37.16 min) as compared to the NS group (158.69 ± 23.64 min) (p < 0.001). The maximum height of the sensory block attained was similar in both groups (p = 0.239) (Table 2). The block level was assessed bilaterally and was similar on both sides.

The postoperative VAS scores were comparable at 0h, 8h and 12h in both groups. However, at 4h and 24h, the DX group demonstrated significantly lower pain scores compared to the NS group (p = 0.001, p = 0.0001 respectively). The mean duration of analgesia or time to the requirement of first additional postoperative analgesia in both groups could not be calculated, as 10 patients (34.4%) in the DX group and 2 patients (6.89%) in the NS group did not require any additional postoperative analgesia in the first 24h after surgery. However, the time to requirement of first additional postoperative analgesia was significantly longer in the DX group. The requirements of additional analgesia with both diclofenac and tramadol were higher in the NS group (Table 2). To adjust for multiple comparisons and control the Family-Wise Error Rate (FWER) in the secondary endpoints (onset time of sensory and motor block, duration of motor block, maximum level of sensory block achieved, postoperative pain scores at 24h, time to requirement of first additional postoperative analgesic, diclofenac consumption over the first 24h and tramadol consumption over the first 24h), Bonferroni correction was applied. The Bonferroni correction (α = 0.00625) revealed that only 4 of the 8 secondary outcomes remained statistically significant i.e., the duration of motor block, postoperative pain scores at 24h, time to first postoperative analgesic and diclofenac consumption over 24h. The rest, including tramadol consumption (p = 0.027), were not significant after adjustment, even though they may have appeared so at the unadjusted α = 0.05 level.

**Figure 3:**
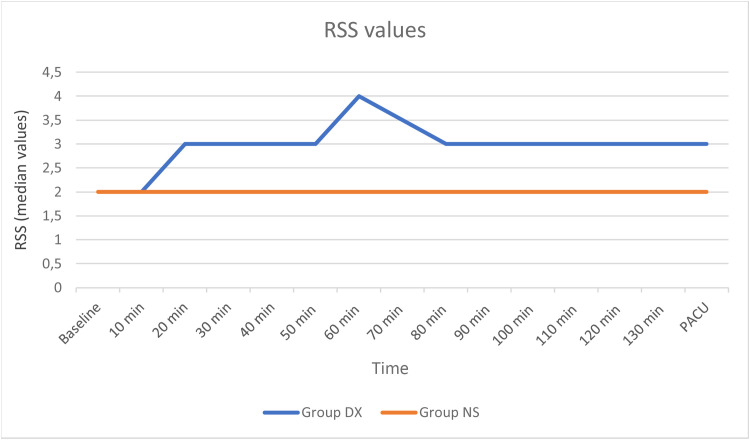
The RSS values were significantly higher in the DX group from 20 min onwards till PACU arrival. RSS, Ramsay Sedation Scores.

The RSS values observed were found to be significantly higher in Group DX from 20 min after the beginning of surgery up to arrival in the PACU (Fig. 3). However, no patient had a RSS > 4/6 indicating excessive sedation.

**Figure 4:**
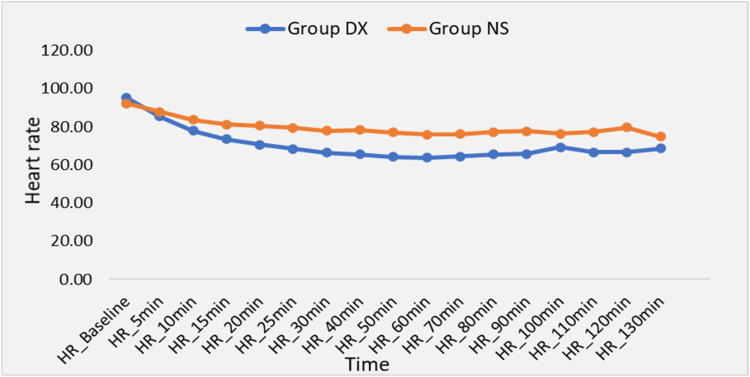
Intraoperative Heart Rate (HR).

**Figure 5:**
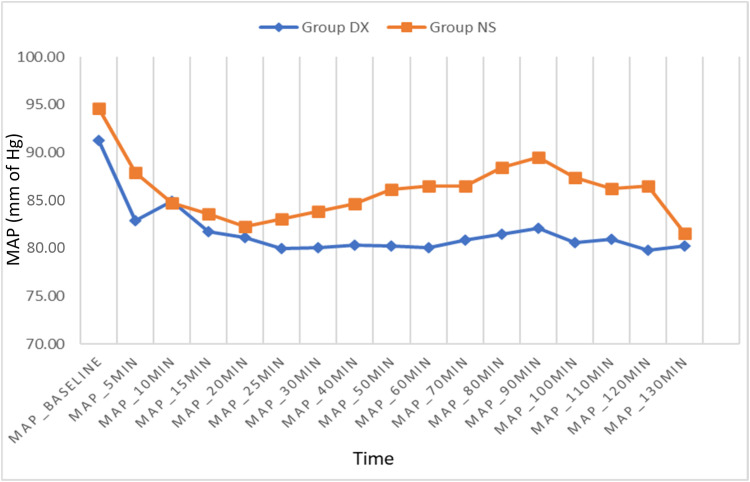
Intraoperative Mean Arterial Pressure (MAP).

HR during surgery was significantly lower in the DX group from 20 to 90 min following administration of spinal anesthesia (p < 0.05) (Fig. 4). The mean HR during surgery was 70.32 ± 8.81 bpm in the DX group and 80.41 ± 12.55 bpm in the NS group (p = 0.001). The Mean Arterial Pressure (MAP) measured during the surgery was also significantly lower in the DX group from 50 to 100 min following administration of spinal anesthesia (p < 0.05) (Fig. 5). The mean value of intraoperative MAP was 81.52 ± 7.08 mmHg in the DX group and 86.04 ± 7.89 mmHg in the NS group (p = 0.026). SpO_2_ showed no significant differences between the groups.

The incidence of bradycardia was significantly higher in the DX group as compared to the NS group. Four (13.79%) patients in the DX group developed bradycardia and received IV atropine 0.5 mg, while no patient in the NS group had bradycardia (p = 0.038). Three patients in the DX group had hypotension as compared to two patients in the NS group (p = 0.640). The hypotension in most cases responded to IV fluids and only one patient in both the groups required a vasopressor. Also, the study drug infusion did not require interruption in any patient. There was no statistically significant difference observed between the groups regarding other side effects such as nausea and vomiting, headache, and excessive sedation. Furthermore, none of the patients needed assistance with ventilation using a bag-mask or endotracheal intubation (Table 3). When asked to guess their group allocation (intervention, control, or unsure) as part of the blinding assessment, we found that the distribution of responses did not differ significantly from chance, with most participants choosing “unsure”. These findings suggest that blinding was successfully maintained throughout the study.

**Table 3:** Incidence of any side effects.

Side effect	Group DX (n = 29)	Group NS (n = 29)	Pearson Chi Square	p-value
Hypotension	3 (10.34%)	2 (6.89%)	0.219	0.640^a^
Bradycardia	4 (13.79%)	0 (-)	4.296	0.038^a^
Nausea	2 (6.89%)	1(3.44%)	0.352	0.553^a^
Vomiting	1(3.44%)	2 (6.89%)	0.352	0.553^a^
Headache	0 (-)	0 (-)	NA	NA
Excessive sedation	0 (-)	0 (-)	NA	NA

Data is expressed as number of patients (percentage of patients).

NA, Not Applicable.

a Using χ^2^ test.

## Discussion

We found that the time to two-segment regression of sensory block was significantly longer in the DX group (137.03 ± 25.02 min) as compared to the NS group (79.45 ± 11.27 min). The duration of motor blockade was also significantly prolonged in the DX group as compared to NS group (236.34 ± 37.16 min vs. 158.69 ± 23.64 min). The onset time of sensory and motor block and the maximum height of sensory blockade achieved was similar in both groups. Postoperative VAS scores were significantly lower at 4h and 24h after surgery and the requirement of additional diclofenac in the post-operative period was significantly reduced in the DX group.

Recent studies have explored the use of various adjuncts to spinal anesthesia aimed at prolonging the duration of sensory blockade. Dexmedetomidine is a highly selective alpha-2 agonist which acts on presynaptic alpha-2 receptors in the locus coeruleus in the brain stem resulting in sedation and analgesia. Postsynaptic activation in the central nervous system inhibits sympathetic activity, leading to a decrease in HR and BP. It is likely that a dexmedetomidine infusion will activate alpha-2 receptors in the spinal cord, resulting in inhibition of transmission of nociceptive impulses,^7^ thus enhancing the action of local anesthetic agents resulting in prolonged sensory blockade and better pain control after surgery.^14^^,^^15^

Intrathecal dexmedetomidine has been used in several studies to prolong the duration of spinal anesthesia with hyperbaric bupivacaine.^16^, ^17^, ^18^ Recent studies have found that the duration of spinal anesthesia can be similarly prolonged by the simultaneous administration of IV dexmedetomidine.^6^, ^7^, ^8^, ^9^^,^^19^, ^20^, ^21^, ^22^, ^23^, ^24^, ^25^, ^26^, ^27^, ^28^, ^29^, ^30^, ^31^, ^32^, ^33^, ^34^ These studies have used various different doses and dose combinations of dexmedetomidine. IV dexmedetomidine prolongs the duration of spinal block primarily by enhancing central analgesia by reducing sympathetic outflow and inhibiting nociceptive transmission. It seems to have a synergistic effect with local anesthetics by systemic absorption and indirect central pain modulation rather than direct action at the level of the spinal cord. By decreasing spinal cord blood flow, it may slow down the clearance of the local anesthetic from the subarachnoid space. The sedation and analgesia provided can mask the waning of the spinal block, thus giving the impression of longer duration.

Some studies have used a single bolus dose of 0.5 mcg.kg^-1^,^6^^,^^18^^,^^25^^,^^30^, ^31^, ^32^ while others have used a bolus of 1 mcg.kg^-1^.^7^^,^^19^^,^^25^^,^^33^ However, a higher incidence of bradycardia and hypotension was observed in some of these studies.^19^^,^^33^ Subsequently investigators studied the use of a bolus followed by infusion of dexmedetomidine to prolong the duration of sensory blockade of spinal anesthesia. The use of a 1 mcg.kg^-1^ bolus followed by an infusion of 0.2 to 0.6 mcg.kg^-1^.h^-1^ resulted in a similar prolongation of sensory blockade but a higher incidence of bradycardia and hypotension.^20^, ^21^, ^22^, ^23^ There are very few studies on the effect of IV dexmedetomidine given as a 0.5 mcg.kg^-1^ bolus followed by a continuous infusion of 0.5 mcg.kg^-1^.h^-1^ throughout the surgery on prolongation of duration of sensory blockade of spinal anesthesia.^7^, ^8^, ^9^ The incidence of side effects has not been reported to be significant with this dose schedule.

We observed that the time to two-segment regression of sensory blockade was 137.03 ± 25.02 min in patients in the DX group and 79.45 ± 11.27 min in the NS group. Kavya et al.^7^ reported a two-segment regression time of sensory blockade of 166.2 ± 26.7 min compared to 133.2 ± 28.2 min in the control group when using an IV bolus of 0.5 mcg.kg^-1^ followed by an infusion of 0.5 mcg.kg^-1^.h^-1^ for 1h. Harsoor et al.^8^ reported time for two-segment regression as 111.52 ± 30.9 min (DX group) vs. 53.6 ± 18.22 min (NS group) when a bolus of IV dexmedetomidine 0.5 mcg.kg^-1^ over 10 min before subarachnoid block was followed by a continuous infusion of 0.5 mcg.kg^-1^.h^-1^ throughout the surgery. Using similar doses, Bhirud et al.^9^ reported a two-segment regression time of sensory blockade of 152.3 ± 18.7 mins, which was similar to what we found.

In earlier studies, a 0.5 mcg.kg^-1^ IV bolus resulted in a time to two-segment regression ranging from 106.67 ± 45.5 min to 148.54 ± 20.66 min.^6^^,^^18^^,^^30^, ^31^, ^32^ A 1 mcg.kg^-1^ bolus of dexmedetomidine resulted in prolongation of two-segment regression time to 171.43 ± 22.89 min up to 178.2 ± 21.8 min,^7^^,^^33^^,^^34^ but with a significant incidence of bradycardia. The use of a 1 mcg.kg^-1^ bolus followed by an infusion resulted in a duration of 130.33 ± 14.49 min to 142.5 ± 2.32 min.^20^^,^^21^^,^^23^ But again, with a higher incidence of bradycardia.

We found that the duration of motor block also showed a significant difference between the two groups. Patients in the DX group had a prolonged duration of motor blockade compared to the NS group (236.34 ± 37.16 min vs. 158.69 ± 23.64 min). Investigators who used the same dosing schedule as ours reported similar findings.^7^, ^8^, ^9^ We observed that the onset time of sensory block and motor block was similar in both groups. This was similar to the observations made in several previous studies.^7^^,^^19^^,^^23^^,^^26^^,^^31^^,^^32^ In contrast, some studies^8^^,^^22^ have reported a faster onset of sensory block in the DX group. These conflicting results could be attributed to factors such as variations in study protocols or methodological differences in assessing the onset of sensory block.

We observed that the maximum height of sensory blockade achieved was similar in both groups. This was again similar to several previous studies.^8^^,^^25^^,^^26^^,^^31^^,^^32^ A few investigators^20^^,^^22^ reported a significant difference in the maximum level of sensory block attained between the dexmedetomidine and the control groups. This may be because of the higher bolus dose of dexmedetomidine administered, i.e., 1 mcg.kg^-1^ unlike the 0.5 mcg.kg^-1^ dose in our study.

As both spinal anesthesia and use of dexmedetomidine reduce sympathetic outflow, the simultaneous administration of both may result in significant bradycardia and hypotension. We observed that MAP was significantly lower in the DX group from the 50^th^ to 100^th^ min and HR was significantly lower from the 20^th^ to 90^th^ min. The hemodynamic response to dexmedetomidine infusion is influenced by both the dosage and the infusion rate. Dexmedetomidine reduces HR and MAP due to a diminished central sympathetic outflow as well as the sedative and anxiolytic actions of dexmedetomidine. We found that the incidence of bradycardia was significantly higher in the DX group as compared to the NS group, which is similar to findings in earlier studies.^19^^,^^20^^,^^22^ However, an incidence of 13.79% is clinically significant, which suggests that dexmedetomidine infusion should be used cautiously in patients with baseline bradycardia or when high levels of neuraxial blockade with consequent sympathetic blockade are required. Although MAP was significantly lower in the DX group, the incidence of hypotension was similar in both groups which is again similar to previous studies.^26^

Intraoperatively, and on arrival in the PACU, patients in the DX group were more sedated as compared to the control group. Dexmedetomidine induces sedation by binding to alpha-2 receptors in the locus coeruleus. This area, along with the dorsal raphe, comprises key central neural structures where it acts producing sedation and analgesia.^35^ Dexmedetomidine is unique in its ability to cause conscious sedation. The fact that RSS scores did not exceed 3/6 in any patient at any time supports this.

We found that postoperative VAS scores were comparable at 0h, 8h and 12h. However, at 4h and 24h, the DX group demonstrated significantly lower pain scores compared to the NS group. The similar VAS scores on arrival in the PACU could be because surgeries were of < 2h duration and it is likely that there was a residual effect of spinal anesthesia. The similarity in VAS scores at 8h and 12h between the DX and NS groups can be attributed to the fact that most of the patients in the NS group had received their first dose of additional analgesic by then. The VAS scores at 24h were significantly lower in the DX group as perhaps, an overall better pain control had been achieved. Although a 1-point reduction (on a 10-point VAS) is usually not considered clinically meaningful,^36^^,^^37^ this should be interpreted keeping in mind that the NS group was also receiving more postoperative analgesics.

All patients in our study received injection Paracetamol 1g IV on arrival in PACU and then henceforth every 8h. Ten patients (34.4%) in the DX group and 2 patients (6.89%) in the NS group did not require any additional postoperative analgesia in the first 24h after surgery and the requirement of additional analgesics in the post-operative period was significantly reduced in the DX group as compared to the NS group. Reduced requirement of postoperative analgesia is beneficial as it reduces the side effects associated with the use of both opioid and nonopioid analgesics.

With intravenous dexmedetomidine, we achieved a sensory block duration ranging from 102 to 184 min and a motor block duration of 174 to 316 min. This duration appears optimal for patients undergoing elective lower limb orthopedic procedures of approximately 1–2 hours under spinal anesthesia. The prolongation of both sensory and motor blockade obviates the need for intraoperative epidural catheterization, which is advantageous given the increasing use of peripheral nerve blocks for postoperative analgesia ‒ techniques that offer several benefits over epidural analgesia in orthopedic settings.

An extended sensory block facilitates a smoother transition into the postoperative period by reducing early pain and enhancing patient comfort and satisfaction. This, in turn, lowers the immediate postoperative opioid requirement, decreases the need for rescue analgesics, and lessens the workload for recovery room staff ‒ aligning well with Enhanced Recovery After Surgery (ERAS) protocols. Moreover, many lower limb orthopedic surgeries are associated with significant postoperative pain. In such cases, prolonged sensory blockade can delay or reduce the need for systemic opioids, minimizing opioid-related adverse effects such as nausea, sedation, and respiratory depression. These benefits are particularly relevant for elderly or opioid-sensitive patients, for whom opioid-sparing strategies are clinically advantageous.

In addition to improving patient outcomes, longer blocks may also enhance surgical team satisfaction by preventing early breakthrough pain in the Post-Anesthesia Care Unit (PACU). However, overly prolonged sensory or motor blockade may delay mobilization, voiding, and discharge ‒ especially in frail or elderly patients ‒ potentially increasing the risk of urinary retention, pressure ulcers, or falls. Early mobilization is critical in orthopedic surgery to minimize the risk of venous thromboembolism and to support functional recovery. If the motor block persists excessively, it may interfere with physiotherapy, hinder ambulation, and prolong hospital stay. Patients with residual motor weakness may also be unaware of limb instability, increasing fall risk during early mobilization.

Overall, intraoperative use of intravenous dexmedetomidine offers distinct clinical advantages in lower limb orthopedic surgeries by prolonging spinal anesthesia in a controlled and effective manner, provided careful consideration is given to the duration of motor block and its impact on postoperative recovery.

### Limitations of the study

Only ASA I/II patients undergoing surgical procedures of < 2h duration were recruited in this study. Our results cannot be extrapolated to ASA III/IV patients undergoing prolonged surgical procedures under spinal anesthesia. The sample size of the study may not have been adequate to detect significant differences in the secondary outcomes. We assessed the effects of dexmedetomidine on spinal anesthesia with only a fixed dose of 2.5 mL of 0.5% hyperbaric bupivacaine in spinal anesthesia and one fixed loading (bolus) and maintenance dose of dexmedetomidine. Hence, the dose response relationship of dexmedetomidine and the duration of spinal anesthesia could not be discussed. As there was no long-term patient follow-up, we cannot comment on rebound hyperalgesia or other delayed side effects.

## Conclusion

Intravenous dexmedetomidine (0.5 mcg.kg^-1^ bolus followed by 0.5 mcg.kg^-1^.h^-1^ infusion) prolonged the duration of sensory and motor blockade and improved postoperative analgesia in patients undergoing lower limb orthopedic surgery under spinal anesthesia, with minimal adverse effects. Further studies are warranted to evaluate optimal dosing strategies and safety in higher-risk populations.

## Data availability statement

The datasets generated during and/or analyzed during the current study are available in the SciELO Data repository - https://doi.org/10.48331/SCIELODATA.N9KIZX. Any aditional data will be made available upon reasonable request to the corresponding author.

## Authors' contributions

Simran Chahal: Conceptualization; Methodology; Software. Anju R. Bhalotra: Conceptualization; Methodology; Data curation; Writing-original draft preparation. Rahil Singh and Snigdha Singh: Methodology; Visualization; Investigation. Shweta Dhiman: Supervision; Software; Validation.

## Funding

This research did not receive any specific grant from funding agencies in the public, commercial, or not-for-profit sectors.

## Conflicts of interest

The authors declare no conflicts of interest.
